# Identification and Phylogenetic Analysis of *Tityus pachyurus* and *Tityus obscurus* Novel Putative Na^+^-Channel Scorpion Toxins

**DOI:** 10.1371/journal.pone.0030478

**Published:** 2012-02-15

**Authors:** Jimmy A. Guerrero-Vargas, Caroline B. F. Mourão, Verónica Quintero-Hernández, Lourival D. Possani, Elisabeth F. Schwartz

**Affiliations:** 1 Laboratório de Toxinologia, Departamento de Ciências Fisiológicas, Universidade de Brasília, Brasília, Distrito Federal, Brazil; 2 Instituto de Biotecnología, Universidad Nacional Autónoma de México, Cuernavaca, Morelos, Mexico; Institut national de la santé et de la recherche médicale - Institut Cochin, France

## Abstract

**Background:**

Colombia and Brazil are affected by severe cases of scorpionism. In Colombia the most dangerous accidents are caused by *Tityus pachyurus* that is widely distributed around this country. In the Brazilian Amazonian region scorpion stings are a common event caused by *Tityus obscurus*. The main objective of this work was to perform the molecular cloning of the putative Na^+^-channel scorpion toxins (NaScTxs) from *T. pachyurus* and *T. obscurus* venom glands and to analyze their phylogenetic relationship with other known NaScTxs from *Tityus* species.

**Methodology/Principal Findings:**

cDNA libraries from venom glands of these two species were constructed and five nucleotide sequences from *T. pachyurus* were identified as putative modulators of Na^+^-channels, and were named Tpa4, Tpa5, Tpa6, Tpa7 and Tpa8; the latter being the first anti-insect excitatory β-class NaScTx in *Tityus* scorpion venom to be described. Fifteen sequences from *T. obscurus* were identified as putative NaScTxs, among which three had been previously described, and the others were named To4 to To15. The peptides Tpa4, Tpa5, Tpa6, To6, To7, To9, To10 and To14 are closely related to the α-class NaScTxs, whereas Tpa7, Tpa8, To4, To8, To12 and To15 sequences are more related to the β-class NaScTxs. To5 is possibly an arthropod specific toxin. To11 and To13 share sequence similarities with both α and β NaScTxs. By means of phylogenetic analysis using the Maximum Parsimony method and the known NaScTxs from *Tityus* species, these toxins were clustered into 14 distinct groups.

**Conclusions/Significance:**

This communication describes new putative NaScTxs from *T. pachyurus* and *T. obscurus* and their phylogenetic analysis. The results indicate clear geographic separation between scorpions of *Tityus* genus inhabiting the Amazonian and Mountain Andes regions and those distributed over the Southern of the Amazonian rainforest. Based on the consensus sequences for the different clusters, a new nomenclature for the NaScTxs is proposed.

## Introduction

Scorpion venoms are a rich mixture of several components, among which are free nucleotides, lipids, biogenic amines, proteins and peptides. Scorpion peptides can be classified into disulfide-bridged (DBPs) and non-disulfide-bridged peptides (NDBPs). The NDBPs exhibit diverse biological functions, including bradykinin-potentiating, antimicrobial, hemolytic and immune-modulating activities [1]. DBPs are considered the main molecules responsible for the neurotoxic effects observed in scorpion envenoming as they affect ion-channels function of excitable and non-excitable cells. The best known DBPs are those specific for Na^+^ or K^+^ channels (NaScTxs and KTxs, respectively) [2], [3].

The NaScTxs are long-chain peptides with 55–76 amino acid residues and cross-linked by three or four disulfide bridges [4], [5], [6]. They are divided into two main classes: the α-NaScTxs, that slow or inhibit the current inactivation of Na^+^ channels and prolong the action potential by binding to receptor site 3 of Na^+^ channels, and the β-NaScTxs, mostly from New World (North and South America) scorpions, which typically shift the voltage dependence of activation to more hyperpolarized potentials and reduce the peak current amplitude by binding to receptor site 4 of Na^+^ channels [7], [8].

Alpha and beta-NaScTxs share a conserved three-dimensional structure consisting of a βαββ topology (see review [9]). The α-NaScTxs can be further divided into three sub-groups: ‘α-classic’, that are very toxic to mammalians; ‘anti-insect α-NaScTxs’, which are highlyspecific to Na^+^-channels of insects; and ‘α-like’, which act on Na^+^-channels of both insects and mammalians [3], [10]. The β-NaScTxs are divided into four sub-groups: anti-mammalian β-toxins that are highly toxic to mammals; anti-insect excitatory toxins; anti-insect depressant; and β-like toxins that are highly active on both insect and mammalian Na^+^-channels [11], [12], [13].

The NaScTxs are responsible for the most dangerous neurotoxic effects observed during human envenoming caused by scorpion sting, also denominated scorpionism, which is a public health problem around the world that mainly affects children and has a complex and controversial treatment [14]. The geographical variability in scorpion species and in their venom composition has become extremely important to the production of effective anti-venoms [15]. The scorpionism in Central and South America is mainly caused by two genera of scorpions: *Centruroides* and *Tityus*, both belonging to the Buthidae family. The genus *Tityus* has a wide distribution from Costa Rica to Northern Argentina [16] and is responsible for many severe casesofscorpionism in Brazil and Colombia [17], [18]. In Colombia, the most dangerous accidents are caused by *Tityus pachyurus* [18], in Venezuela by *T. discrepans* and *T. zulianus* [19] and in Brazil by *T. obscurus* at Amazonian region [20]. These four species inhabit the Northern part of South America and are isolated by the Amazon Basin from the other species of *Tityus* genus as: *T. serrulatus*, *T. bahiensis*, *T. stigmurus* and *T. fasciolatus* from Brazil and *T. trivittatus* from Argentina. This may suggest the existence of a phylogenetic relationship between the NaScTxs of the venoms from scorpions distributed in the Northern South America, as well as an evolutive differentiation drove by biogeographic separation within South America.

In Colombia, *Tityus pachyurus* is distributed along the Magdalena River valley in cities and villages between 400 to 1500 m of altitude [21], [22]. Its venom has a lethal dose in mice of 4.8 mg/kg, and two of its toxins and one acidic peptide were previously indentified, respectively: Tpa1, which acts on K^+^-channels, Tpa2, which modulates Na^+^-channels, and Tpa3, of unknown function. Its proteomic analysis showed at least 104 compounds with distinct molecular masses [23].

Another Buthidae scorpion with medical importance from Northern South America is *Tityus obscurus* Gervais, 1843 – a senior synonym of both *Tityus paraensis* Kraepelin, 1896 and *Tityus cambridgei* Pocock, 1897 [24], which is responsible for many poisoning cases in humans in the Brazilian Amazonian region [20]. To the same species of scorpion three different systematic names were attributed. Here we decided to follow the earliest taxonomic classification: *T. obscurus*. Analysis of the venom from this scorpion demonstrated the existence of at least 102 distinct peptide components, of which about 25% have their N-terminal sequences determined [25] and, thus far, the complete primary structure of six peptides are known. Three of them are K^+^-channel specific toxins: Tc1, Tc30 and Tc32 [26], [27]. The other three toxins act on Na^+^-channels: Tc49b, a non α-scorpion toxin that at 100 nM concentration abolishes almost completely the Na^+^-current in rat cerebellum granular cells [28]; Tc48a, a peptide that affects Na^+^-permeability in F-11 cells lines in culture at nanomolar concentrations [25]; and Tc48b/Tc49a, which affects Na^+^-permeability in pituitary GH3 cells in culture, in a similar mechanism as those reported for the α-scorpion toxins [29]. The trivial names of these toxic peptidespreviouslyused were designed from the abbreviation of the genus (T) and the species (c, from *cambridgei*), followed by a number that corresponds to the elution time (in minutes) from HPLC separation [26]. Since in this communication the senior name of *Tityus obscurus* was adopted, the trivial names are now named as “To”followed by the number of the peptide.

This study reports for the first time the molecular cloning of the NaScTx precursors from *T. pachyurus* and *T. obscurus* scorpions by means of cDNA library constructions of their venom glands, presenting new putative NaScTxs and also the precursor sequences of some Na^+^-channel peptides already described from previous proteomic researches with these two scorpion venoms [23], [25], [28], [29]. We also have employed proteomic approach, by means of chromatographic, mass spectrometry and automated Edman degradation analyses, to isolate some NaScTxs from *T. pachyurus* and *T. obscurus* scorpion venoms and to determine, with the support of the cDNA libraries, their primary sequences and molecular masses. A complete phylogenetic analysis with these NaScTxs and other Na^+^-channel toxins from*Tityus* genus species registered in public database was performed to infer whether the geographic separation between the scorpions of *Tityus* genus caused by the Amazon Basin led to evolutionary changes in these sequences.

## Materials and Methods

### 1. Venom source and chemicals

Nineteen specimens of *T. pachyurus* were collected in the municipality of Mesitas de la Escuela, in the state of Cundinamarca in Colombia, under the *Corporación Autónoma Regional de Cuandinamarca* license number 1096, and 23 specimens of *T. obscurus* were collected in the state of Amapá, Brazil, under the *Instituto Brasileiro do Meio Ambiente e dos Recursos Renováveis* (IBAMA) license number 048/2007-CGFAU. They were kept alive in the laboratory at the University of Brasília, in individual terrariums and fed fortnightly with cockroaches and received water *ad libitum*. Once a month the venom was extracted by electrical stimulation of the last metassomal segment (telson), collected in 1.5 mL tubes, diluted in deionized water and centrifuged at 10,000× *g* for 15 min, discarding the pellet. Protein concentrations in the soluble venom and in purified venom fractions were estimated by the absorbance at 280 nm [30]. After protein quantification, the samples were dried under vacuum and stored at −20°C. All solvents and chemicals used in this study were analytical grade and deionized water was used throughout.

### 2. Purification procedures

One mg of dried venom from *T. pachyurus* and *T. obscurus*, separately,was solubilized in 200 µL deionized water, centrifuged at 14,000× *g* for 15 min at room temperature and the supernatant was submitted to high performance liquid chromatography (HPLC), using an analytical C18 RP column (250 mm×10 mm) (Phenomenex, USA). The venom molecules were separated using a linear gradient applied from solution A (0.12% trifluoroacetic acid –TFA– in water) to 60% solution B (0.10% TFA in acetonitrile) during 60 min and eluted at a flow rate of 1 mL/min, with detection at 216 and 230 nm. Fractions were individually and manually collected, vacuum dried and stored at −20°C until use. The fractions of interest were further separated by HPLC using optimized conditions based on their retention times and percentage of B solution.

### 3. Mass spectrometry analysis

#### 3.1. ESI-MS

The chromatographic fractions of interest from *T. pachyurus* venom were reconstituted in a 50% acetonitrile/1% acetic acid solution and directly applied into a Finnigan LCQ^DUO^ ion trap mass spectrometer (San Jose, CA) using a Surveyor MS syringe pump delivery system at a flow rate of 10 µL/min. The fractions were splitted to allow only 5% of the sample to enter the nanospray source. The operation voltage used was 2.00 kV and the coaxial nitrogen flow was adjusted as needed for optimum sensitivity. All spectra were obtained in the positive ion mode. Data acquisition was performed on Xcalibur Windows NT PC data system.

#### 3.2. MALDI-TOF MS

Molecular mass analysis from *T. obscurus* venom fractions was performed on an UltraFlex III MALDI TOF/TOF mass spectrometer (Bruker Daltonics, Germany) in the positive linear mode. Chromatographic fractions were reconstituted in deionized water at variable concentrations and dissolved in an α-cyano-4-hydroxycinnamic acid matrix solution (1∶3, v∶v), spotted in triplicate onto a MALDI target plate and dried at room temperature for 15 min. Calibration of the system was performed using the Peptide Calibration Standard for Mass Spectrometry calibration mixture (up to 4000 Da mass range, Bruker Daltonics). Spectra were processed with MassLynx™ 3.5 (Manchester, UK) and FlexAnalysis 2.4 (Bruker Daltonics, Germany).

### 4. Amino acid sequence determination

Amino acid sequencing of purified peptides was performed by the automated Edman degradation method on a PPSQ-23 protein peptide sequencer (Shimadzu Co., Japan). For the To5 toxin from *T. obscurus* venom the amino acid sequence was also determined by MALDI – *in source decay* (ISD) utilizing 1,5-diaminonaphthalene matrix solution [31]. Similarity searches were performed using BLAST (www.ncbi.nlm.nih.gov/blast) and FASTA 3 (www.ebi.ac.uk/fasta).

### 5. Construction of cDNA libraries and gene cloning

The cDNA libraries were separately constructed from total RNA extracted from a single telson of a *T. pachyurus* and a *T. obscurus* scorpion, as previously described [4]. The scorpions were milked 5 days before RNA extraction. The RNAs of each species were extracted using the SV Total RNA Isolation System Kit (Promega, Madison, WI). The full-length cDNA libraries were prepared by means of the Creator SMART cDNA Library Construction kit (CLONTECH Lab., Palo Alto, CA). cDNA inserts were cloned into the plasmids pDNR-LIB digested by restriction enzymes Sfi I. The recombinant plasmids were transformed into electrocompetent *Escherichia coli* DH5α. For the polymerase chain reaction (PCR) of both cDNA libraries the universal oligonucleotides T7 and M13 were used as primers. Selected plasmids with cDNAs>400 bp were isolated using alkaline lyses method, and single-pass sequencing of the 5′-termini was conducted with the primer T7 using an automatic sequencer (Model 3100, Applied Biossystems, Foster City, CA) according to the manufacturer's instructions.

### 6. Bioinformatics analysis

To extract the high quality sequence region, the ESTs were subjected to the Phred program as previously described [32] with the window length set to 100 and the standard quality to 20. The CrossMatch program was used to remove vector sequences. ESTs that shared an identity of >95 out of 100 nucleotides were assembled in contiguous sequences with the CAP3 program [33]. All these bioinformatics analysis were simultaneously run at the http://www.biomol.unb.br/ site using default setup. The *T. pachyurus* and *T. obscurus* cDNA sequences were searched against nr public database using blastx and blastn algorithms http://www.ncbi.nlm.nih.gov/blast/ with an e-value cut-off set to <10^−5^ to identify putative functions of the new ESTs. All sequences were examined for existence of signal peptides using the SignalP 3.0 program http://www.cbs.dtu.dk/services/SignalP/and the pro-peptide cleavage site was determined from the known start site of previously characterized mature toxins. The theoretical molecular masses of the putative mature peptides were calculated in the online service PeptideMass http://www.expasy.ch/tools/peptide-mass.html. The nucleotide sequences obtained in this work are deposited in EMBL Nucleotide Sequence Database numbers HE585239 to HE585243 for *T. pachyurus* and HE585224 to HE585238 for *T. obscurus*.

### 7. Alignment and phylogenetic analysis

Mature putative sodium toxins from *T. pachyurus* and *T. obscurus* were compared with other sodium scorpion toxins of the *Tityus* genus registered in the UniProt database http://www.uniprot.org/. The toxin AaHIT4 (UniProtKB P21150), from the African scorpion *Androctonus australis*, was selected as the outgroup [34]. Multiple sequence alignments were performed by CLUSTAL_X 1.83 software followed by manual adjustment [35]. This result was subsequently used to build the phylogenetic analysis and consensus sequences. In the sequence matrix, all positions containing gaps and missing data were eliminated. The Maximum Parsimony method with 500 Bootstrap replications [36] and Close-Neighbor-Interchange algorithm model [37] on MEGA 5 software was used in the reconstruction of the phylogenetic tree [38]. The analysis involved 66 amino acid sequences.

## Results

### 1. Molecular cloning

**Figure 1:**
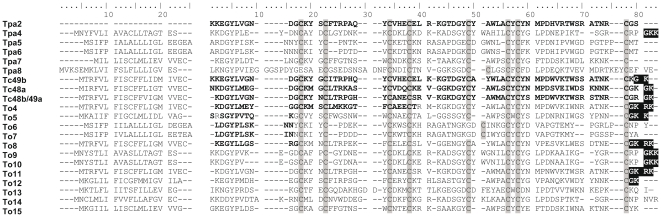
Multiple sequence alignment of Sodium-channel toxins from *T. pachyurus* (Tpa) and *T. obscurus* (To). The amino acid sequences already described in literature are showed in bold. Predicted amino acid sequences are shown with the putative signal peptides in the left and the putative mature toxins in the right, with the identified C-terminal prosequences shaded in black. Cys residues from the mature peptides are shaded in grey.

The titers of the non-amplified cDNA libraries obtained were 1.4×10^4^ cfu/mL with95% recombinant clones for *T. pachyurus* and 1.7×10^4^ cfu/mL with 95% recombinant clones for *T. obscurus*. After the analysis from the venomous gland cDNA libraries, the independent clones from *T. pachyurus* and *T. obscurus* were submitted to bioinformatic analysis to remove vector and poor quality sequences, and a total of 5 (in 3 contigs and 2 singlets) nucleotide sequences of high quality from *T. pachyurus* and 15 (in 14 contigs and 1 singlet) from *T. obscurus* were identified as precursors of putative modulators of Na^+^-channels, with a mean read length of 254nucleotides (ranging from 243 to 266nucleotides). These transcripts have in average a signal peptide with 20–22 amino acid residues and a mature segment which shares several amino acid residues characteristics of the typical NaScTxs as well as the conformation of four disulfide bridges, as presented in Fig. 1, which also includes the previously reported NaScTx Tpa2 [23]. One mature peptide (Tpa4) from *T. pachyurus* and ten (Tc49b, Tc48a, Tc48b/Tc49a, To4, To5, To8, To9, To10, To11 and To12) from *T. obscures* might present the C-terminal amidated, which is a conserved posttranslational modification in many NaScTxs previously described [3]. When the nucleotide sequences terminate with Gly followed by basic residues it is assumed that these carboxy-terminal residues are removed by a carboxypeptidase, after which the remaining peptide would show the most C-terminal amino acid amidated [39]. All sequences were submitted to blastn and blastx searches against nr database and an e-value<10^−5^ was used as cut-off for confidential homologue detection. The complete sequences of all precursors of the putative NaScTx transcripts, described here below, are deposited in the data bank as indicated in Material and methods (section 2.6).

#### 1.1. Sodium channel toxins from *T. pachyurus*

The five NaScTx transcripts from *T. pachyurus* cDNA library were named Tpa4, Tpa5, Tpa6, Tpa7 and Tpa8 (Fig. 1). These trivial names come from the abbreviation of the genus, with the first letter capitalized (T), and the first two letters in lowercase (pa) indicating the specific epithet, and the following number used corresponds to the chronological order of the description of a new toxic peptide, as first suggested by Becerril and collaborators [40]. An equivalent nomenclature was also adopted for the*T. obscurus* putative toxins (“To” and the corresponding number).

The Tpa4 can be classified as a classical α toxin by its high identity (82%) with the TbTx5 (UniProtKB P0C5K8), identified at transcript level from the Brazilian scorpion *T. bahiensis* [41], and 81% identity with alpha-mammalian toxin Ts3 (UniProtKB P01496) purified from the venom of another Brazilian scorpion *T. serrulatus* [42]. This putative toxin has a theoretical average molecular mass of 7405.0 Da (Table 1) and is constituted by 64 amino acid residues with 8 cysteines, which are likely to form four disulfide bridges. By similarity, its C-terminal is enzymatically cleaved at GKK and amidated.

**Table 1:** Toxins from *T. pachyurus* and *T. obscurus* and their average molecular masses and retention times (RT).

Toxin	Previous name	MM		RT (min)	Reference
		Theoretical	Experimental		
**Tpa2**	Tpa2 (P84631.1)	7522.5	7522.0	36.2	[23]
**Tpa4**	-	7405.4	-	-	This work
**Tpa5**	-	7342.4	-	-	This work
**Tpa6**	-	7294.3	-	-	This work
**Tpa7**	-	6985.0	-	-	This work
**Tpa8**	-	8481.2	-	-	This work
**Tc49b**	Tc49b (P60214)	7404.5	7405.6	36.6	[28], this work
**Tc48a**	Tc48a (P60212)	7319.3	7318.3	35.1	[25], this work
**Tc48b/Tc49a**	Tc48b/Tc49a (P69213)	7385.4	7385.2	36.6	[29], this work
**To4**	Tc54 (P60215)	7254.6	7253.2	40.0	[28], this work
**To5**	-	6937.7	6937.1	45.4	This work
**To6**	Tc43 (P84685.1)	7266.3	7266.0	34.3	[25], this work
**To7**	Tc50 (P84688.1)	7074.1	7073.0	37.9	[25], this work
**To8**	-	7050.0	-	-	This work
**To9**	-	7155.2	-	-	This work
**To10**	-	6940.9	-	-	This work
**To11**	-	7154.2	-	-	This work
**To12**	-	7171.2	-	-	This work
**To13**	-	8054.0	-	-	This work
**To14**	-	7953.0	-	-	This work
**To15**	-	7195.1	-	-	This work

Swiss-Prot accession numbers are present when available.

Both Tpa5 and Tpa6 putative toxins have 66 amino acid residues, theoretical molecular masses of 7342.4 and 7294.3 Da (Table 1), respectively, and 8 cysteines each. These two Tpa toxins have 67% identity with the putative alpha neurotoxin TdNa9 (UniProtKB C9X4K7) identified at transcript and protein levels, and 66% with the putative neurotoxin TdNa10 (UniProtKB C9X4K8), that has been evidenced at transcript level, both from the Venezuelan scorpion *T. discrepans* [43].

Analysis of the Tpa7 toxin showed that it has 62 amino acid residues, theoretical molecular mass of 6985.0 Da (Table 1), 8 cysteines, and can be classified as a β-NaScTx. This peptide shares 78% identity with the beta-neurotoxin Tz2 (UniProtKB Q1I165), identified at transcript and protein levels from the Venezuelan scorpion *T. zulianus* [44], and with the TdNa6 (UniProtKB C9X4K4)from *T. discrepans*, also evidenced at both levels [43].

**Figure 2:**
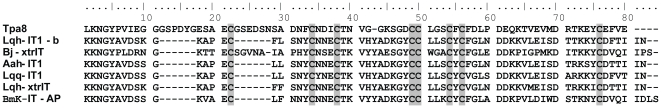
Multiple sequence alignment of Tpa8 with other anti-insect β excitatory NaScTxs from the Old World. Tpa8 putative toxin from *T. pachyurus*, LqhIT1b from *Leirus quinquestriatus hebraeus*, Bj-xtrIT from *Buthotus judaicus*, AahIT1 from *Androctonus australis*, LqqIT1 from *Leiurus q.quinquestriatus*, Lqh-xtrIT from*Leirus q. hebraeus* and BmK IT-AP from *Mesobuthus martensii*.

The putative Tpa8 toxin, which is constituted by 79 amino acid residues and 8 cysteines, with a theoretical average molecular mass of 8481.2 Da (Table 1), presented 43% identity with the toxin LqhIT1b (UniProtKB P68722), identified at protein level from *Leiurus quinquestriatus hebraeus* venom [45]. The Tpa8 toxin is the first register of an anti-insect excitatory β-toxin from scorpions of the Buthidae family from the New World (Fig. 2) and, by similarity, presents an important structural feature, with the fourth disulfide bridge shifted when compared to the other β-toxins. This structural modification is an important and exclusive feature of the anti-insect excitatory β-NaScTxs [12].

#### 1.2. Sodium channel toxins from *T. obscurus*

**Figure 3:**
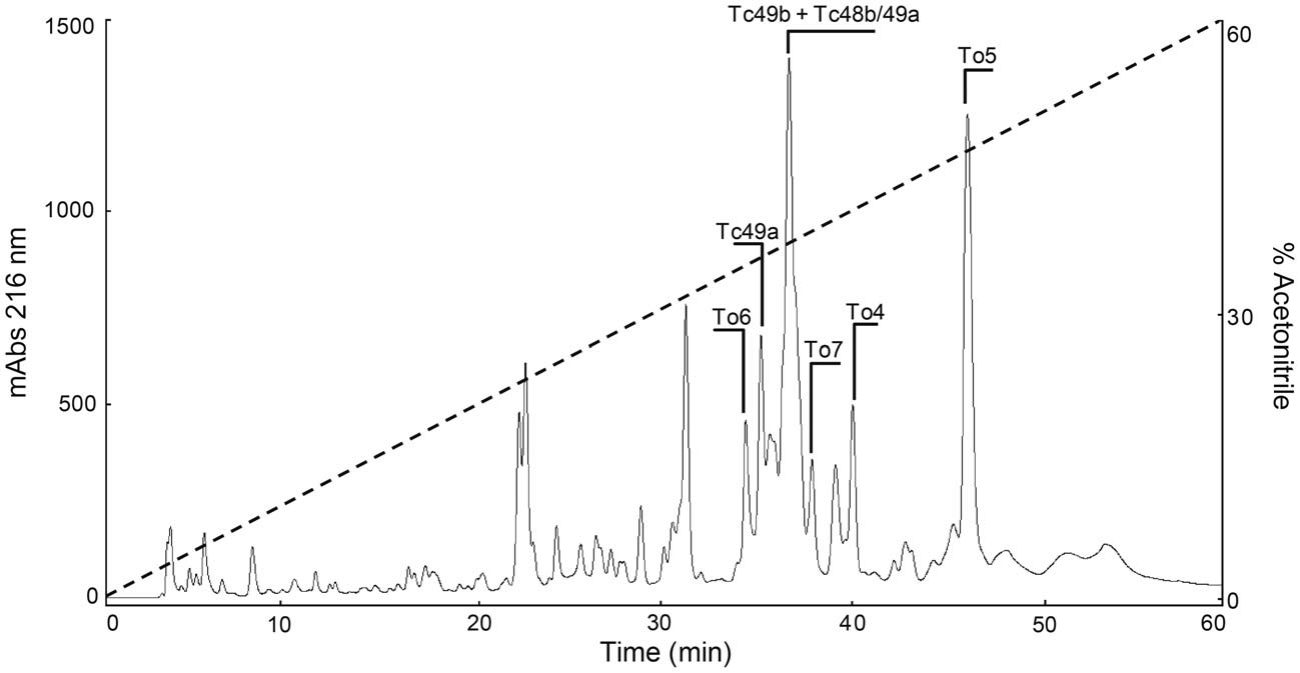
High performance liquid chromatography separation of 1.0 mg soluble venom from *T. obscurus*. This was performed in an analytical C18 reversed phase column equilibrated with solution A (water in 0.1% TFA), using a gradient from 0 to 60% solution B (acetonitrile in 0.12% TFA) over 60 min, with a flow rate of 1 mL/min and absorbance at 216 nm. Fractions labeled in the chromatogram (Tc49b to To7) were detected at both protein and transcript levels.

Fifteen distinct sequences from *T. obscurus* encode for NaScTxs (Fig. 1). The mature peptides corresponding to three of them were previously reported: Tc49b [28], Tc48a [25] and Tc48b/Tc49a [29]. As we propose the adoption of the senior name *Tityus obscurus* instead of *T. cambridgei*, these toxin names should be replaced to To1, To2 and To3, respectively, where the following number actually used corresponds to the description order for the Na^+^-channel toxins. However, their actual trivial names were kept in order to avoid any further confusion. Analysis of these three transcript sequences, described for the first time in the present study, revealed that the two last amino acid residues (-GK) from their C-terminal were enzymatically removed, so these peptides assumed their mature form. The signal peptides from Tc49b, Tc48a and Tc48b/Tc49a transcripts have 20 amino acid residues each, from which 15 are equal in all these precursor sequences showing they are highly conserved (Fig. 1). These three peptides are indicated in the chromatographic profile obtained by the separation of 1.0 mg soluble venom from *T. obscurus* (Fig. 3). Tc49b and Tc48b/Tc49a eluted together, as previously reported [28], [29], at 36.6 min.

N-terminal amino acid sequences and experimental molecular masses corresponding to the mature peptides To4, To6 and To7 (showed in bold in Fig. 1) were previously described [25], [28] and early named as Tc54, Tc43 and Tc50, respectively (Table 1). Their complete putative sequences were described for the first time in the present study. The theoretical molecular masses for these putative sequences, already considering the C-terminal processing, are equivalent to their experimental molecular masses [25], confirming the complete sequences (Table 1). Analysis conducted with these transcripts showed that these precursors encode mature peptides with 62 to 65 amino acid residues, each with 8 cysteines which are likely to form four disulfide bridges. To4 mature toxin, which eluted at 40.0 min (Fig. 3) and has an experimental molecular mass of 7253.2 Da and a C-terminal posttranslational modification (-GKRK removal), shares a representative identity (77%) with the putative beta-neurotoxin Td11 (UniProtKB Q1I173), identified at transcript level from *T. discrepans* [44] and with the putative beta-neurotoxins TdNa7 (UniProtKB C9X4J8) (79%) and Td3 (UniProtKB Q1I177) (77%), both identified at protein level from *T. discrepans* venom [43], [44]. Both To6 and To7, with experimental molecular masses of 7266.0 and 7073.0 Da, respectively (Table 1), present high identity (70 and 69%, respectively) with the putative neurotoxin TdNa10 (UniProtKB C9X4K8), evidenced at transcript level from *T. discrepans* and 61 and 63%, respectively, with the putative neurotoxin TdNa9 (UniProtKB C9X4K7), identified at protein level from the same scorpion [43]. To6 and To7 eluted at 34.3 and 37.9 min, respectively, as showed in the chromatogram (Fig. 3).

Analysis conducted with the transcript To5 showed that it encodes for a mature peptide with 62 amino acid residues found in the scorpion venom and reported for the first time in the present study (Fig. 1). This peptide, which eluted at 45.4 min in the crude venom HPLC fractionation (Fig. 3), presented 62% identity with Ardiscretin (UniProtKB P0C1X7), from *T. discrepans* scorpion venom, a single polypeptide composed by 61 amino acid residues with an amidated cysteine residue at the C-terminal end, packed by four disulfide bridges [43]. The first 10 amino acid residues from To5 sequence were similar to that described for Tc66 [25], which eluted at 45.18 min. These two peptides present very close molecular masses, however, Arg2 in To5 is replaced by Tyr2 in Tc66. Considering their elution times in HPLC fractionations (which were performed using identical procedures), and taking into account their molecular masses, these differences might be due to the presence of a homologous form eluting from the column at the same percentage of acetonitrile. The average molecular mass experimentally determined for To5 was 6937.1 Da, very close to the theoretical molecular mass of 6937.7 Da (Table 1). The first 40 amino acid residues from To5 were obtained by Edman degradation and the complete sequence was confirmed by MALDI-ISD, utilizing 1, 5-diaminonaphthalene matrix solution (data not shown). The same sequence was obtained from the results of the cDNA library. The activity of this peptide has not been determined yet.

The To8 putative mature peptide presents a theoretical molecular mass of 7050.0 Da (Table 1) if considered the expected C-terminal processing (-GKRK removal) (Fig. 1). Its first ten N-terminal amino acid residues were equal to those described for the peptide Tc61 described by Batista and collaborators [25], though this last peptide presents an experimental molecular mass of 7105.0 Da. Therefore, due to the different molecular masses, To8 and Tc61 are probably different peptides. After the chromatographic fractioning of *T. obscurus* venom performed in the present study (Fig. 3), it was identified a peptide with 7107.8 Da, eluted at 42.8 min, equivalent to Tc61, which elutes at 42.64 min [25]. However, as this peptide was not obtained in sufficient amount to be sequenced, the equivalence still has to be analyzed. The putative peptide To8 shares considerable identity (58–62%) with the putative beta-neurotoxins Td7 (UniProtKB Q1I164), Td10 (UniProtKB Q1I176), Td11 (UniProtKB Q1I173), Td6 (UniProtKB Q1I167) and Td9 (UniProtKB Q1I178), evidenced at transcript level [44], and Td1 (UniProtKB Q1I180), Td3 (UniProtKB Q1I177) and Td2 (UniProtKB Q1I179), evidenced at protein level from *T. discrepans* [43], [44].

The putative sequences To9 to To15 (Fig. 1) had no equivalent toxins found in the chromatographic profile until now, and, for this reason, the experimental molecular masses could not be determined (Table 1). The To9 mature peptide has an expected molecular mass of 7155.2 Da and presents 98% identity with the putative alpha-neurotoxin TdNa8 (UniProtKB C9X4K6), evidenced at transcript level from *T. discrepans* [43]. The signal peptides of both putative sequences comprise the first 19 residues at the N-terminal sequence and share 89% identity, with only two different amino acids. The mature peptides from these two putative sequences, which comprise 66 amino acid residues, have only one amino acid different (Ala21 in To9 is replaced by Glu21in TdNa8) (see Table S1 in Supporting Information). Similar to TdNa8, To9 is thought to be post-translationally processed to give a mature peptide of 63 amino acids, in which the C-terminal amino acid proline is amidated (Fig. 1). The three residues after the Pro in the precursor are GKK, which are cleaved and the glycine residue provides the amine group for amidation of Pro. To9 also presents 73% identity with TbTx5 (UniProtKB P0C5K8), a putative alpha-neurotoxin evidenced at transcript level from the scorpion *T. bahiensis* [41], for which an equal processing mechanism is also expected. To10 sequence shares with To9 an equal signal peptide and also the same C-terminal processing, with –GKK removal after Pro (Fig. 1). Its putative mature sequence presents 6940.9 Da and 62 amino acid residues, with only five residues different to To9.

The To11 mature sequence presents 93% identity with the alpha-neurotoxin Tc48b/Tc49aand 90% identity with the putative beta-neurotoxins Tz1 (UniProtKB Q2NME3) and Td4 (UniProtKB Q1I174), identified at transcript and protein levels from the Venezuelan scorpions *T. zulianus* and *T. discrepans*, respectively [44], [46]. Its signal peptide has 20 amino acid residues and, by similarity, it is expected to contain a C-terminal processed peptide by removal of the four last residues after Cys (-GKRK) (Fig. 1). Assuming this maturation process is correct and that the peptide is forming 4 disulfide-bridges, the expected molecular mass for To11 mature peptide should be 7154.2 Da (Table 1).

Analysis conducted with the transcript To12 showed that, after an expected C-terminal processing (-GK removal) and considering a 20 amino acids signal peptide, this precursor encodes for a peptide with 62 amino acid residues, with 8 cysteines which are likely to be forming 4 disulfide bridges (Fig. 1), and with a theoretical molecular mass of 7171.2 Da (Table 1). It presents high identity (85%) with the Tb2-II (UniProtKB P60276) neurotoxin, an active toxin against both mammals and insects evidenced at protein level from *T. bahiensis* venom [47], and 82% with the beta-neurotoxin Ts2 (toxin III-8) (UniProtKB P68410), from *T. serrulatus* venom [48], [49], and its homologous Tst2 (toxin III-8 like) (UniProtKB P68411), from *T. bahiensis* venom [40].

The first 9 and 11 amino acid residues from To13 and To14 mature sequences, respectively, were equal to that from Tc40 (UniProtKB P84683) and Tc41 (UniProtKB P84684) [25], however, the theoretical and experimental molecular masses were significantly different, even considering different C-terminal processing and, as these putative sequences were grouped into contigs with 3 and 9 reads, respectively, we disregard the possibility of erroneous sequences. The To13 transcript has a signal peptide with 18 amino acid residues and a mature peptide with 71 amino acids (Fig. 1), and a theoretical molecular mass of 8054.0 Da (Table 1). The To13 mature toxin presents 43% identity with the putative beta-neurotoxin LmNaTx1 (UniProtKB D9U297) and 42% identity with the putative alpha-neurotoxin LmNaTx21.1 (UniProtKB P0CI53), both evidenced at transcript level from *Lychas mucronatus* Asian scorpion [50]. The To14 transcript contains a signal peptide with 19 amino acids and a mature peptide with 70 amino acid residues (Fig. 1), and an expected molecular mass of 7953.0 Da (Table 1). It presents 61% identity with Pg8 (UniProtKB B7SNV8), a toxin evidenced at protein and transcript levels from *Parabuthus granulatus* scorpion and able to generate protective antibodies in mice [51].

The To15 precursor contains 83 amino acid residues (Fig. 1). Its putative signal peptide (19 residues) is removed and the resulting 64 amino acid peptide has a theoretical molecular mass of 7195.1 Da (Table 1).To15 mature toxin shows significant sequence identity with the putative beta-neurotoxins TdNa6 (UniProtKB C9X4K4) (79%), evidenced at protein and transcript levels from *T. discrepans* [43], and Tz2 (UniProtKB Q1I165) (77%), evidenced at transcript level from *T. zulianus* venom gland [44].

### 2. Phylogenetic analysis

**Figure 4:**
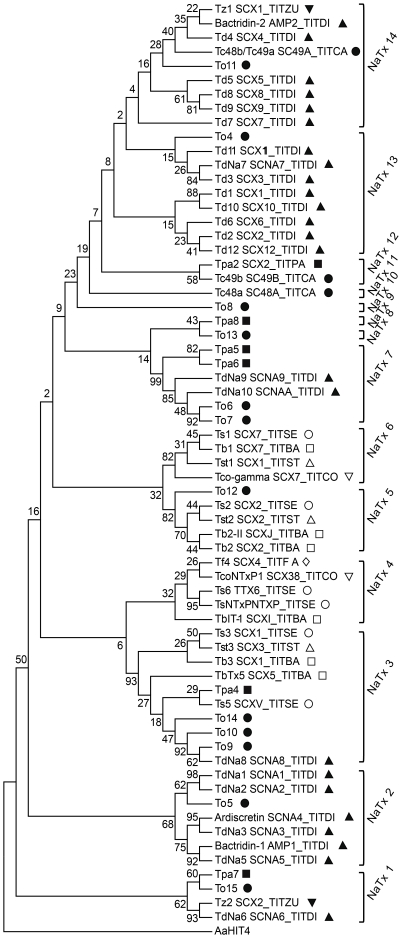
Phylogenetic analysis of NaScTxs from *T. pachyurus*, *T. obscurus* and other scorpions from *Tityus* genus. Filled symbols indicate toxins from scorpions inhabiting the Northern region of the Amazon Basin: square for *T. pachyurus*, circle for *T. obscurus*, triangle for *T. discrepans*, and inverted triangle for *T. zulianus* toxins. Open symbols indicate toxins from scorpions inhabiting the Southern part of the Amazon Basin: square for *T. bahiensis*, triangle for *T. stigmurus*, inverted triangle for *T.costatus*, circle for *T. serrulatus*, and rhombus for *T. fasciolatus* toxins.

Sixty five peptides or deduced peptides from genes of known scorpions of the genus *Tityus* demonstrated or supposed to be specific for Na^+^-channels were used to build the molecular phylogenetic analysis by the Maximum Parsimony (MP) method presented in Fig. 4.It includes seventeen new NaScTx sequences, five from *T. pachyurus* and twelve from *T. obscurus* (this communication), together with Tpa2 and three NaScTxs already described from *T. pachyurus* and *T. obscurus*, respectively, and 44 known NaScTx sequences from other *Tityus* genus species. The bootstrap consensus tree inferred from 500 replicates was taken to represent the evolutionary history of the taxa analyzed [36]. Branches corresponding to partitions reproduced in less than 50% bootstrap replicates are collapsed. The percentage of replicate trees in which the associated taxa clustered together in the bootstrap test (500 replicates) is shown next to the branches [36]. The MP tree was obtained using the Close-Neighbor-Interchange algorithm [37] with search level 2 in which the initial trees were obtained with the random addition of sequences (10 replicates). There were a total of 47 positions in the final dataset.

**Figure 5:**
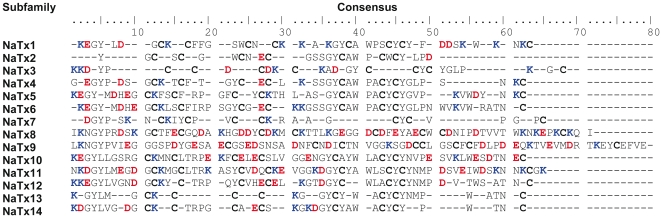
Consensus sequences for the 14 proposed subfamilies of NaTxs from scorpions of the genus *Tityus*. Amino acid sequences were obtained by classical proteomic approach and/or predicted by cDNA library constructions from scorpion venom glands. Acidic and basic residues are shown in red and blue, respectively. Cys residues are shown in bold.

Figure 4 shows the results of a rooted phylogenetic tree, where it was possible to group the Na^+^-channel toxins identified from *Tityus* genus scorpion venoms into 14 subfamilies (NaTx1 to NaTx14), which were also used to form the consensus sequence groups (Fig. 5). (For details, see Table S1 in Supporting Information).

The first cluster, NaTx1, shows a relationship between the toxins Tpa7 from *T. pachyurus*,To15 from *T. obscurus*, Tz2 (UniProtKB Q1I165) from *T. zulianus* and TdNa6 (UniProtKB C9X4K4) from *T. discrepans*. All these toxins present high sequence similarity with β-NaScTxs. In the second cluster, NaTx2, a branch is formed by Ardiscretin (UniProtKB P0C1X7), TdNa1 (UniProtKB C9X4J9), TdNa2 (UniProtKB C9X4K0), TdNa3 (UniProtKB C9X4K1), TdNa5 (UniProtKB C9X4K3) and Bactridin-1 (UniProtKB P0CF39) from *T. discrepans* and To5 from *T. obscurus*. Due to the sequence similarity with Ardiscretin, it is expected that these toxins are also specific for arthropods. All these toxins form the clusters named NaTx1 and NaTx2, which consensus sequences are presented in Fig. 5 and are from scorpions inhabiting the Northern part of South America.

The NaTx3 group was formed by ten NaScTxs from which five toxins – Tpa4 from *T. pachyurus*, To9, To10 and To14 from *T. obscurus* and TdNa8 (UniProtKB C9X4K6) from *T. discrepans* – belong to the species distributed inthe Northern partof the Amazon river, and the other five toxins – Ts3 (UniProtKB P01496) and Ts5 (UniProtKB P46115) from *T. serrulatus*, Tst3 (UniProtKB P0C8X5) from *T. stigmurus* and Tb3 (UniProtKB P56608) and TbTx5 (UniProtKB P0C5K8) from *T. bahiensis* – are from species distributed in the Southern region of the Amazon Basin. This was the only branch in our phylogenetic relationship hypothesis with the same proportion of toxins belonging to *Tityus* genus species which inhabit the Southern and Northern areas of the Amazon Basin. All sequences from this group share high sequence similarities with the α-NaScTxs and form the consensus sequence showed in Fig. 5.

The next cluster, NaTx4, comprises Ts6 (UniProtKB P45669) and TsNTxP (UniProtKB O77463) toxins, from *T. serrulatus*, TcoNTxP1 (UniProtKB Q5G8A8) from *T. costatus*, TbIT-1 (UniProtKB P60275) from *T. bahiensis* and Tf4 (UniprotKB P83435) from *T. fasciolatus*. These four species inhabit the Southern part of the Amazon Basin, with highly conserved domains (Fig. 5).

In the next branch, two main groups, NaTx5 and NaTx6, were formed with most Na^+^-channel toxins from *Tityus* species inhabiting the Southern region of South America. In the NaTx5 group, the closely related toxins Ts2 (UniProtKB P68410) from *T. serrulatus*, Tst2 (UniProtKB P68411) from *T. stigmurus*, Tb2 (UniProtKB P56609) and Tb2-II (UniProtKB P60276)from *T. bahiensis* were grouped with To12 toxin from *T. obscurus*, which is the only NaScTx in this group belonging to a scorpion from the Amazonian rainforest. In the next cluster, NaTx6, it is noteworthy to see the close relationship between four NaScTxs identified in scorpion venoms that inhabit the Southern area of the Amazonian rainforest: Tco-gamma (UniProtKB Q5G8B8) from *T. costatus*, Ts1 (UniProtKB P15226) from *T. serrulatus*, Tb1 (UniProtKB P56611) from *T. bahiensis* and Tst1 (UniProtKB P56612) from *T. stigmurus*. These peptides have high percentage of sequence identity with β-NaScTxs, showing in the consensus sequences high conserved domains along the whole sequences (Fig. 5).

From NaTx7 to NaTx14, there were only NaScTxs from scorpions inhabiting the Northern region of South America, strengthening the proposal of the geographic separation caused by the Amazon Basin. The NaTx7 group included exclusively Na^+^-channel toxins from *T. pachyurus* (Tpa5 and Tpa6), *T. obscurus* (To6 and To7) and *T. discrepans* (TdNa9 and TdNa10 – UniProtKB C9X4K7 and C9X4K8, respectively). All these sequences present sequence similarity with α-NaScTxs.

Due to their particular primary sequences, the next four peptides – To13, Tpa8, To8 and Tc48a (UniProtKB P60212) – formed independent branches that were labeled NaTx8,NaTx9,NaTx10 and NaTx11, respectively. Besides being in the same branch, To13, from *T. obscurus*, and Tpa8, from *T. pachyurus*, were classified into different subfamilies as they share only 29% identity. All these peptides present sequence similarity with β-NaScTxs, although Tc48a, previously described, has a mechanism of action similar to the typical α-NaScTxs [25].

The toxins Tpa2 (UniProtKB P84631) and Tc49b (UniProtKBP60214) from *T. pachyurus* and *T. obscurus*, respectively, formed the next cluster, named NaTx12. These two toxins, with highly conserved domains (Fig. 5), share sequence similarity with β-NaScTxs.

The next branch separates the NaTx13 group from the NaTx14. The NaTx13 clusteris formed by eight toxins from *T. discrepans*: Td11 (UniProtKB Q1I173), TdNa7 (UniProtKB C9X4J8), Td3 (UniProtKB Q1I177), Td1 (UniProtKB Q1I180), Td10 (UniProtKB Q1I176),Td6 (UniProtKB Q1I167),Td2 (UniProtKB Q1I179) and Td12 (UniProtKB Q1I172); and the To4 toxin from *T. obscurus*. The NaTx14 cluster, with a higher conserved domain (Fig. 5), comprises six toxins from *T. discrepans*: Bactridin-2 (UniProtKB P0CF37), Td4 (UniProtKB Q1I174), Td5 (UniProtKB Q1I169), Td8 (UniProtKB Q1I163), Td9 (UniProtKB Q1I178) andTd7 (UniProtKB Q1I164); two toxins from *T. obscurus*: To11 and Tc48b/Tc49a (UniProtKB P60213); and one toxin, Tz1 (UniProtKB Q2NME3), from *T. zulianus*. Similar to Tc48a [25], Tc48b/Tc49atoxin also presents a mechanism of action similar to the typical α-NaScTxs [29], besides presenting sequence similarity with β-NaScTxs, such as all the other toxins from these two clusters.

## Discussion

In this work, we have employed a transcriptomic approach to investigate Na^+^-channel putative peptides. They are supposed to be modulators of Na^+^-channel function and were obtained from both *T. pachyurus* and *T. obscurus*scorpions. In addition, a proteomic approach was used in order to obtain their primary sequence and molecular masses. Data published previously and public data banks with these venom peptides were also used in order to complete these parameters. Phylogenetic analysis with *Tityus* NaScTxs was done to infer whether the geographic separation between the scorpions of *Tityus* genus living in the North and South of the Amazon region led to evolutionary changes in these sequences.

Using the cDNA libraries obtained from one venomous gland from *T. pachyurus* and one from *T. obscurus*, a considerable number of clones were obtained and sequenced: 4 different nucleotide sequences of high quality from *T. pachyurus* and 15 from *T. obscurus* were identified as precursors of putative modulators of Na^+^-channels. It is important to notice that only one scorpion of each species was used, thus the variations on structure sequences found cannot be attributed to intra-species variations. Although this information does not represent the total number of NaScTxs present in their venom, since we constructed a non-amplified library, it could be expected that clone number reflects the actual prevalence of a given transcript. The representativeness of the components was more effectively demonstrated for *T. obscurus*, as many NaScTxs present in its cDNA library were also detected at protein level in this study and also in previous proteomic analysis conducted with the venom of this species [25], [28], [29]. The experimental molecular masses of many peptides identified in *T.obscurus* venom are equivalent to the theoretical molecular masses of putative mature toxins (Table 1). In the last years, transcriptomic and proteomic approaches have been used to explore the molecular composition of venoms, showing the great number of different toxin families [52]. Considering these two approaches, however, the transcriptomic strategy shows to be more effective in the description of new venom peptide sequences [53], [54], including even the atypical venom molecules that can hardly be isolated by conventional methods [55].

Novel trivial toxin names were suggested in this communication for the NaScTxs partially described from *T. obscurus* scorpion venom (Table 1), as they carry information about the junior name *Tityus cambridgei* Pocock, 1897 [24]. Tc54, which was partially described by Batista and collaborators [28], was renamed To4. The To5 denomination was given to a new toxin, evidenced at protein and transcript levels in the present study and which presents considerable identity with Ardiscretin, a toxin from *T. discrepans* venom which was shown to be specific for invertebrates (crickets, triatomides, crabs and squids), but non-toxic to mice at the dose assayed [56]. The Tc43 and Tc50 [25] were renamed To6 and To7, and the other putative NaScTxs from *T. obscurus*, with no equivalent toxins detected at protein level until now, were named To8 to To15 (Table 1).To avoid further misunderstandings, *T. pachyurus* NaScTxs nomenclature has begun with Tpa4 in the present study, even though Tpa1 and Tpa3 are not Na^+^-channel toxins [23].

An advance in the study and discovery of novel peptide toxins from scorpion venoms using the proteomic and transcriptomic approaches [53], [57], [58] has increased the need for development of a rational nomenclature for naming these toxins in order to facilitate future cataloguing and analysis (see review [59]). Since the novel nomenclature proposed by King and collaborators [59]is not universally accepted we decided to maintain the general denomination of α- and β-toxins, adding however the results of our phylogenetic analysis, that certainly can help a better way of classifying these toxic peptides (see below). The nomenclature proposed by Tytgat and collaborators [2] for the potassium channel scorpion toxins, based on a primary sequence alignment of the toxins and considering the cysteine and other highly conserved residues, was successfully adopted by researches and it is in constant update, thus permitting the inclusion of new K^+^ toxin families.

Based on the currently used nomenclature for the K^+^-channel scorpion toxins [2] and in order to avoid many denominations for a single peptide, as it occurs with many *T. serrulatus* toxins [60], we propose a new unified nomenclature for the long-chain NaScTxs. The 65 peptide sequences from *Tityus* scorpion venoms used in our phylogenetic analysis were clustered in 14 subfamilies named NaTx1 to NaTx14 (Fig. 4), based on the alignment of cysteine residues and other highly conserved domains (Fig. 5). In the Supporting Information (Table S1) included in this manuscript, the individual members of each of the subfamilies are presented with their amino acid sequences, percentage of identity with the first described toxin from each subfamily, main features, and corresponding references. This classification method permits the continuous addition of new Na^+^-channel scorpion toxins in the presented groups, by the addition, in the chronological order of description, of a new cardinal number after the subfamily number (see the fourth column of Table S1 in Supporting Information), and even the inclusion of new subfamilies, as different NaScTxs are discovered. The proposed nomenclature can be adopted by toxins or peptides identified by both methods, direct protein isolation and transcript levels, and do not abolish the trivial names used until now, which are also important to facilitate remembering the biological source of the toxins, as many of them are associated with the scorpion species denomination.

As mentioned above, the new putative toxins identified in this study present sequence similarities with other previously identified toxins and for this reason, some are classified as α (Tpa4, Tpa5, Tpa6, To6, To7, To9, To10 and To14) and β NaScTxs (Tpa7, To4, To8, To12 and To15), although some special cases should be highlighted.The Tpa8 toxin was classified as an anti-insect excitatory β-NaScTx for its structural similarity with the classical excitatory toxins from buthid scorpions from the Old World (Fig. 2). Into the NaScTxs, the β excitatory are the largest peptides in length, with 70–76 amino acid residues, and share a common scaffold comprising an α-helix and three stranded anti-parallel β-sheet stabilized by four disulfide bridges. However, in the excitatory toxins the fourth disulfide bridge is shifted when compared to the other β toxins [12], [61]. By similarity with the Bj-xtrIT toxin (UniProtKB P56637), from the Asian buthid scorpion *Buthotus judaicus* (Fig. 2), we could infer two active domains: the pharmacophore, which in Tpa8 consists of Glu25, Asp26, Asp36 flanked by Phe33 and Val41, and the second amino acid cluster in the functional surface, which is formed by hydrophobic residues positioned on the C-terminal (Phe77 and Val78).

Tc49b toxin [28] presents 64 amino acid residues and 7405.6 Da (Table 1) and shares 82% identity with Tpa2, a β-NaScTx from *T. pachyurus* scorpion venom [23]and 69% with the putative beta-neurotoxins Tz1 and Td4, identified at transcript and protein levels from the Venezuelan scorpions *T. zulianus* and *T. discrepans*, respectively [44], [46]. The actual electrophysiological data with Tc49b is not sufficient to allow speculations on its fine mechanism of action, but as it does not seem to change the inactivation mechanism of Na^+^-channels, it is suggestive that this toxin does not act as a typical α-NaScTx [28]. Tc48a toxin, which presents 65 amino acid residues and 7318.3 Da [25] (Table 1), has considerable identity (63–65%) with the putative beta-neurotoxins Td11, Td7, Td1, Td12, Td3 and Td10 from *T. discrepans* [43], [44], but its mechanism of action is similar to that from the typical α-NaScTxs [25]. In a similar way, Tc48b/Tc49atoxin, which presents 64 amino acid residues and 7385.2 Da [29] (Table 1), shares high identity (90%) with the beta-neurotoxins Tz1 and Td4 from *T. zulianus* and *T. discrepans*, respectively [44], [46], but it affects Na^+^-permeability in pituitary GH3 cells in a similar mechanism as those reported for the α-scorpion toxins [29]. To8 putative mature peptide, which shares 58 to 62% identity with many putative beta-neurotoxins from *T. discrepans* (see Results, section 3.1.2), also has considerable identity with Tc49b (62%), Tc48b/Tc49a (58%) and Tc48a (55%). Contrary to Tc49b and to all other putative beta-neurotoxins which showed identity with To8, these latter two toxins present a mechanism of action similar to that from the typical α-NaScTx [25], [28], [29]. To11 and To13 putative toxins also have sequence similarities with both α and β-NaScTxs.

Tc48a and Tc48b/Tc49a toxins and the discovery of several new scorpion peptides assumed to be toxic suggested the existence of some inconsistence on the designation of α-toxins found in the Old World and the β-toxins in the New World (see review [62]). It imposes the question whether it is possible to classify the toxins into alpha or beta classes only by searching against databases without previous acquisition of electrophysiological data. This is currently done when only transcriptomic analysis is performed, without direct measurement of the real biological function, and is important as it contributes to the knowledge of the state of art in scorpion toxins, but not sufficient to determine their real activity.

There are also several peptides with typical long-chain structure that display divergent effects and do not fit strictly into classical α or β classes. AahSTR1, a non-toxic peptide isolated from the North African scorpion *Androctonus australis* Hector, shares sequence similarity with Old World α-toxins, whereas its 3D structure is similar to that from the New World β-toxins [63]. AahIT4, another long-chain peptide isolated from this same scorpion venom, modulates the specific binding of both α- and β-type anti-mammal scorpion toxins to the mammal Na^+^-channel and is also toxic to insects. Therefore, this peptide is a possible phylogenetic link between Old World and New World scorpion toxins [34], and for this reason it was selected as the outgroup in our phylogenetic analysis. BmP09 [64] and KAaH1 [65], from *M. martensii* and *A. australis* Hector, respectively, specifically block K^+^-channels, despite the fact that they are structurally more closely related with other NaScTxs. Another peptide named Birtoxin, which has only three disulfide bonds and shares high homology with *Centruroides* β-toxins, was recently isolated from the South African scorpion *Parabuthus transvaalicus* [66].

Froy and Gurevitz [62] have proposed an evolutionary route for the NaScTxs in which the ancestral β-toxin might have developed into α-toxins before the separation of the continents, which can explain the existence of some α-toxins in South America buthids (e.g. CsEV, Ts3 and Ts5, the two latter clustered in the NaTx3subfamily – Fig. 4) and the large portion of α-toxins in Old World scorpion venoms. In the New World, the ancestral β-toxin has more likely developed into the existing β-toxins (e.g. Cn2, Css II and Ts1, the latter clustered in the NaTx6subfamily – Fig. 4), which predominate in the New World buthid scorpion venoms, and has also given rise to a new group of α-toxins, named by the authors α′-toxins, as they have preserved the β-toxin structure but acquired pharmacological features of α-toxins similar to that from the Old World (e.g. CsEv1, CsEv3). Due to the sequence similarity of the *Tityus* α-toxins to the Old World α-toxins, it is possible that the ancestor that gave rise to this genus existed before the separation of South America from Africa. The ancestral *Tityus* β-toxins might have further developed into the existing β-toxins that actually predominate in these venoms [62].

In the α-NaScTxs, the active surfaces can be identified by two domains: the “core-domain” is formed by residues of the loops connecting the secondary structure elements of the molecule core, which have a chemical nature highly conserved and is predominated by positively charged and hydrophobic residues, and the “NC-domain” comprises five residues (between the 8–12 chain position) and a C-terminal segment formed by a residue-turn in the positions 56–64 [67], [68]. These structural features, common to the Old World α-NaScTxs, are present in the toxins from the NaTx3 cluster (Fig. 4), which includes the new putative toxins Tpa4 from *T. pachyurus* and To9, To10 and To14 from *T. obscurus*, with the “core-domain” formed by acidic and hydrophobic residues and the “NC-domain” more variable in amino acids but with a basic C-terminal tail. This is in agreement with the proposed by Gurevitz and collaborators [61] in which the α-toxins contain a conserved hydrophobic surface and the carboxy-terminal stretch has a limited degree of structural freedom that permits the formation of a variety of bioactive regions. In that cluster it is also observed an important region that is the conserved N-terminal surface constituted by the hydrophilic and basic amino acids Lys1, Lys2, Asp3, Gly/Asp4, Tyr5 and Pro6 (Fig. 5), being these features observed only in the New World α-NaScTxs.

The NaTx7cluster (Fig. 4) is also formed by toxins with high similarity with the α-NaScTxs and all of them are from scorpions that inhabit the Northern of the Amazon region. These toxins share the same active surfaces, owning the “core-domain” and the “NC-domain” postulated by Gordon and collaborators [67] but the “core-domain” in this toxin group is more similar to the “core-domain” from the α-like toxins from the Old World scorpion venom. The N-terminal surface of the toxins from this cluster shares the hydrophilic feature of the N-terminal region of the NaTx3 cluster, but is less variable in relation to the amino acids that confer the basic property. In the NaTx4 cluster (Fig. 4 and Table S1 from Supporting Information), four toxins were previously classified as α-NaScTxs, with only one peptide (TbIT-1) classified as β-NaScTx.

The NaTx1, NaTx2, NaTx5, NaTx6 and NaTx9 to NaTx14clusters (Fig. 4) are closely related with the β-NaScTxs. Cohen and collaborators [69] suggested that the pharmacophore is one of the bioactive surfaces on β-NaScTxs and is associated with the α-helix of the toxin peptide. Another bioactive surface is the C-terminal tail and the loop that links the α-helix with the β-sheet [12], [61]. In the NaTx5, NaTx6 and NaTx14 clusters obtained by the phylogenetic relationship presented here (Fig. 4), the pharmacophore surface is constituted by amino acid residues that are closely related with the amino acids of the anti-mammalian and anti-insect β-NaScTx pharmacophores, suggesting that theseβ-toxins of the genus *Tityus* are also toxic to insects. The clusters NaTx1, NaTx2and NaTx9 to NaTx13 have the amino acid residues of the pharmacophore surface similar to the pharmacophore of the depressant anti-insect β-NaScTxs, although it was demonstrated that the Tc48a toxin, in the NaTx11cluster, presents a β-type structure, but an α-type action on mammals [25],and that the Tpa2 toxin, in the NaTx12 cluster, also presents activity on mammals [23]. In all these clusters related with the β-NaScTxs, the C-terminal surface is variable, strengthening the idea that the C-terminal region in sodium toxins is in constant evolution, process that may have occurred in parallel to the evolutionary changes of target sites in sodium channels [61].

From our analysis, the phylogenetic inference of *Tityus* scorpion NaTxs revealed a strong separation between the species *T. pachyurus*, *T. obscurus*, *T. discrepans* and *T. zulianus* living in the Northern part of the Amazon Basin and those living in its Southern part, as *T. serrulatus*, *T. bahiensis*, *T. stigmurus*, *T. costatus* and *T. fasciolatus*. This separation coincides with the morphoclimatic regions (Amazon, Guyana, Choco, Atlantic Forest, Araucaria, Chacao, Cerrado, Caatinga, Pantanal, Gran Sabana, Llanos, Cerrado-Amazon transition region, Chacao-Amazon transition region and Pacific) of tropical South America, which is one of the most biologically diverse regions on Earth [70], [71].*T. obscurus* is distributed in the Northern part of the Amazon region in Brazil, whereas *T. pachyurus*, *T. discrepans* and *T. zulianus* are localized in the Mountain Andes region, the first in Colombia and the others in Venezuela.

Similarly, the Amazonian region is famous for high biodiversity, the highlands of the transition zone between the Andes and the lowlands of the Amazon Basin show particularly high species diversity. Hypotheses proposed to explain the high levels of diversity in the highlands include repeated parapatric speciation across ecological gradients spanning the transition zone, repeated allopatric speciation across geographic barriers between the highlands and lowlands, divergence across geographic barriers within the transition zone and simple lineage accumulation over long periods of time, which were influent facts in the patters of divergence in frogs of the genus *Epipedobates* [72]. These parameters are also observed in scorpion speciation and divergence. Lourenço [73] postulated that South American tropical scorpions exhibit a high degree of endemism in the Amazon and Atlantic Forest transition, in the Amazon and Choco Forest transition, and also in the Andes region, all areas which appear to be the epicenter of scorpion diversity in the World. This could be observed in the phylogenetic analysis presented here, where the NaScTxs from *T. pachyurus* and *T. obscurus* were mostly grouped with *T. discrepans* and *T. zulianus* NaScTxs (Fig. 4), all species belonging to the Amazon and Andes transition. This statement is consistent with the criteria that *Tityus* reaches its greatest diversity in the Northwestern part of South America with half of the species described from Colombia, Ecuador and Venezuela [19], [74]. The other scorpion species – *T. costatus*, *T. bahiensis*, *T. stigmurus*, *T. serrulatus* and *T. fasciolatus* – belong to the Amazon and Atlantic Forest transition and also to Cerrado biome.

These ecogeographical differences and environmental changes in the South American scorpion habitats not only contributes with the speciation process on scorpion of the *Tityus* genus, but also lead to important diversity of components in scorpion venoms. It is worth mentioning that the diversity in scorpion venoms not only occurs between species, but also into populations of the same species. In the comparative venom gland transcriptome analysis of *Lychas mucrunatus* scorpions from different geographical regions, it was revealed high intraspecific toxic gene diversity and that scorpions evolve to adapt a new environment by altering the primary structure and abundance of venom peptides and proteins [50]. This intraspecific diversity of scorpion venom peptides was also showed in the venom of *Scorpio maurus palmatus* from four geographically isolated localities in Egypt [15].

All these evidences in inter and intraspecific variation of scorpion venoms caused by geographical isolation should be considered in the significant variations of the scorpionism symptoms. In this regard, an investigation addressing phylogeography of *Androctonus* species in Tunisia shows evidence for regional variation in toxins from *A. australis* venom between the two morphological forms *A. a. garzonii* and *A. a. hector*, and between another species of *Androctonus*, suggesting the anti-venom production using both *A. australis* subspecies [75]. Differently, in a recent study made by Amaro and collaborators [76], it was showed that a human antibody fragment (ScFv) specific for the Ts1toxin (UniProtKB P15226) from *T. serrulatus* scorpion venom had a stronger recognition for the Ts1 toxin, for which it was built, but also 60% of recognition for Tc49b from *T. obscurus*, 50% for Tpa2 from *T. pachyurus* and 15% for Cn2 from *Centruroides noxius*. Nevertheless, this result still indicates a difference between these toxins and the geographical separation of the *Tityus* genus species.

The geographical separation inferred in the phylogenetic tree performed in this study can be also observed by the differential clinical manifestations due to a scorpion sting. Scorpion envenomation is an important public health problem in tropical and subtropical zones due to its frequent incidence and potential severity, and the management of some cases can be difficult especially in regions with limited medical facilities, as it happens in several remote areas of the Mountain Andes and Amazon regions [14].

In the envenoming caused by *T. obscurus*, the main observed effects are central neurotoxicity as myoclonia, dysmetria, dysarthria and ataxia, with minimum or no autonomic manifestations [20], [77]. Although *T. obscurus* has an Amazonian distribution and *T. pachyurus*, *T. discrepans* and *T. zulianus*have a Mountain Andes distribution, all these species share the clinical manifestations in the scorpionism cases as central neurotoxicity [20], [78], [79], similar to other species found in the Northwestern of the Amazon Basin, as *T. asthenes* and *T. nematochiurus*, from Colombia, and *T. perijanensis*, from Venezuela, which are phylogenetically related [19], [79] and are also responsible for severe envenomation cases [18], [79]. These clinical manifestations are different to the symptoms caused by the stings from scorpions found in the Southeastern part of Brazil, where the manifestations observed are mainly autonomic with few or no neurotoxic effects [77], [80], [81].

Otherwise, in Venezuela, it was reported in the scorpionism evoked by *Tityus neospartanus* acute pancreatitis and cardiac electrical abnormalities evidenced by premature auricular and ventricular contractions, elevation of the ST segment, depression of the J point, prominent U wave, depression of the ST segment and sinus arrhythmia [82]. These effects are different from those caused by other *Tityus* species from Venezuela and Brazil. In this regard, it has been proposed grouping the scorpion species responsible for severe scorpionism cases in Venezuela into toxinological provinces, based on the clinical consequences of the envenomation, the immunological cross-reactivity of their venoms and their phylogenetic affinity [19].

The phylogenetic separation proposed in the present study might be considered for producing efficient anti-venoms for the scorpionism caused in these different regions of South America. There are several evidences suggesting the existence of a strong biogeographic influence on *Tityus* speciation as well as in the toxinological properties of venoms [74]. Otherwise, there are some evidences that the commercial anti-venoms do not have the same power of neutralization on envenomation caused by *Tityus* species in different regions. *T. discrepans* anti-venom, for example, does not abolish the effect of *T. serrulatus* venom [83] and has a medium to low power against *T. zulianus* and *T. perijanensis* [84]. These observations should be taken into consideration by the diverse countries of the region for the fabrication of anti-venoms.

## Supporting Information

Table S1 **Proposed nomenclature for the 65 members grouped in 14 subfamilies of long-chain NaTxs from** ***Tityus*** **genus scorpions.** Gaps were introduced to improve the alignment. Identical residues of each subfamily are shaded in grey. The percentage identity (% Id.) was calculated using ClustalW algorithm (http://www.ebi.ac.uk/Tools/msa/clustalw2/), considering as 100% the toxin which completes primary sequence was firstly described from each subfamily. In the Characteristics column, three parameters were considered. (i) Protein existence: P for the toxins isolated and partially or completely sequenced from the venom; P* for the toxins which were similar to the putative toxins only by molecular mass comparison; T for the putative toxins evidenced at transcript level. (ii) Function: Arthr, Mice, Ins and Frog for toxins active on arthropods, mice, insects or frog nerve, respectively; Antm for antimicrobial peptides; Immun for an immunogenic protein; and Allerg for a peptide which induced generalized allergic reaction on mice. (iii) Classification: α or β for the toxins with electrophysiological data by means of patch clamp techniques; α′ for the toxins with β-toxin structure but pharmacological features of α-toxins; α for the toxin for which the α-activity was proposed by means of sucrose gap experiments. For (ii) and (iii) parameters, the not underlined items are those predicted by sequence similarity, but not experimentally determined. The toxins from *Tityus pachyurus* (Tpa2, Tpa4, Tpa5, Tpa6, Tpa7 and Tpa8) and *Tityus obscurus* (Tc49b, Tc48a, Tc48b/Tc49a and To4 to To15) are in bold. Ts1, Ts2, Ts3, Ts5, Ts6, TsNTxP are from *T. serrulatus*; Tb1, Tb2, Tb2-II, Tb3, TbTx5 and TbIT-1 are from *T. bahiensis*; Tst1, Tst2 and Tst3 are from *T. stigmurus*; Tco-gamma and TcoNTxP1 are from *T. costatus*; Tf4 is from *T. fasciolatus*; Tz1 and Tz2 are from *T. zulianus*; and Td1 to Td12, TdNa1, TdNa2, TdNa3, TdNa5, TdNa6, TdNa7, TdNa8, TdNa9, TdNa10, Ardiscretin, Bactridin-1 and Bactridin-2 are from *T. discrepans*. The new toxins identified or completely sequenced in the present study are identified by traces in the reference column. Partially sequenced toxins were not taken into account.
(DOCX)
