# Supplementary material for: Identification and Phylogenetic Analysis of Tityus pachyurus and Tityus obscurus Novel Putative Na+-Channel Scorpion Toxins
Source: PLoS One. 2012 Feb 15;7(2):e30478. doi: 10.1371/journal.pone.0030478 (PMC3280238; doi:10.1371/journal.pone.0030478)
Supplement: Table S1 — Proposed nomenclature for the 65 members grouped in 14 subfamilies of long-chain NaTxs from Tityus genus scorpions. Gaps were introduced to improve the alignment. Identical residues of each subfamily are shaded in grey. The percentage identity (% Id.) was calculated using ClustalW algorithm (http://www.ebi.ac.uk/Tools/msa/clustalw2/), considering as 100% the toxin which completes primary sequence was firstly described from each subfamily. In the Characteristics column, three parameters were considered. (i) Protein existence: P for the toxins isolated and partially or completely sequenced from the venom; P* for the toxins which were similar to the putative toxins only by molecular mass comparison; T for the putative toxins evidenced at transcript level. (ii) Function: Arthr, Mice, Ins and Frog for toxins active on arthropods, mice, insects or frog nerve, respectively; Antm for antimicrobial peptides; Immun for an immunogenic protein; and Allerg for a peptide which induced generalized allergic reaction on mice. (iii) Classification: α or β for the toxins with electrophysiological data by means of patch clamp techniques; α′ for the toxins with β-toxin structure but pharmacological features of α-toxins; α for the toxin for which the α-activity was proposed by means of sucrose gap experiments. For (ii) and (iii) parameters, the not underlined items are those predicted by sequence similarity, but not experimentally determined. The toxins from Tityus pachyurus (Tpa2, Tpa4, Tpa5, Tpa6, Tpa7 and Tpa8) and Tityus obscurus (Tc49b, Tc48a, Tc48b/Tc49a and To4 to To15) are in bold. Ts1, Ts2, Ts3, Ts5, Ts6, TsNTxP are from T. serrulatus; Tb1, Tb2, Tb2-II, Tb3, TbTx5 and TbIT-1 are from T. bahiensis; Tst1, Tst2 and Tst3 are from T. stigmurus; Tco-gamma and TcoNTxP1 are from T. costatus; Tf4 is from T. fasciolatus; Tz1 and Tz2 are from T. zulianus; and Td1 to Td12, TdNa1, TdNa2, TdNa3, TdNa5, TdNa6, TdNa7, TdNa8, TdNa9, TdNa10, Ardiscretin, Bactridin-1 and Bactridin-2 are from T. di [file pone.0030478.s001.docx]

Supporting Information

**Table S1. Proposed nomenclature for the 65 members grouped in 14 subfamilies of long-chain NaTxs from *Tityus* genus scorpions.**

| **Subfamily** | | | **Sequence** | **Charact.** | **NaTx - name** | **% Id.** | **Refs** |
| --- | --- | --- | --- | --- | --- | --- | --- |
|  |  | |  |  |  |  |  |
| **1** | Tz2 | | -KEGYLLDKS NGCKRSCFFG STSWCNTECK SKSAEKGYCA WPSCYCYGFS DDSKMWDLKT NKC-- | P*T β | NaTx1.1 | 100 | [1] |
|  | TdNa6 | | -KEGYLLDRS NGCKRSCFFG STSWCNTECK SKSADKGYCA WPSCYCYGFT DDSKMWHLKT NKC-- | PT Arthr β | NaTx1.2 | 93 | [2] |
|  | **Tpa7** | | -KEGYPLDTL NGCKVGCFFG TNSWCNDKCK SKTAAKGYCA WPSCYCYGFT DDSKIWDLKK NKC-- | T β | NaTx1.3 | 77 | – |
|  | **To15** | | GKEGYPLDS- SGCKAGCFFG TNSWCNTECK RKSAAKGYCA WPSCYCYEFT DDSKIWNAKT NKCYK | T β | NaTx1.4 | 77 | – |
|  |  | |  |  |  |  |  |
| **2** | Ardiscretin | | -KNGYIIEPK GCKYSCFWGS STWCNRECKF KKGSSGYCAW PACWCYGLPD NVKIFDYYNN KC | PT Arthr β | NaTx2.1 | 100 | [3] |
|  | TdNa1 | | -RDAYPADWR GCKFSCFWGS SSWCNEECTS LGGSSGYCAW PACWCYGLPD SVRYYNNKCH K- | PT Arthr β | NaTx2.2 | 68 | [2] |
|  | TdNa2 | | -RDAYPADWR GCKPSCPWGS SSWCNEECTS LGGSSGYCAW PACWCYGLPD SVRYYNNKCH K- | T Arthr β | NaTx2.3 | 66 | [2] |
|  | TdNa3 | | -KNGYIIEPK GCKYSCSWGS STWCNRECKF KKGSSGYCAW PACWCYGLPD NVKIFDYYNN KC | PT Arthr β | NaTx2.4 | 98 | [2] |
|  | TdNa5 | | -KDGYIIEHR GCKYSCFFGT NSWCNTECTL KKGSSGYCAW PACWCYGLPD NVKIFDSNNN KC | T Arthr β | NaTx2.5 | 80 | [2] |
|  | Bactridin-1 | | -KDGYIIEHR GCKYSCFFGT NSWCNTECTL KKGSSGYCAW PACWCYGLPD NVKIFDSNNL KC | P Arthr Antm β | NaTx2.6 | 78 | [4] |
|  | **To5** | | SRSGYPVTQK GCVYSCFWGS NWWCNAECTA LGGSSGYCAW PSCWCYSLPD NRNIWGSYPN NC | PT Arthr β | NaTx2.7 | 62 | – |
|  | |  |  |  |  |  |  |
| **3** | | Ts3 | KKDGYPVE-- YDNCAYICWN YDNA-YCDKL CKDKKADSGY CYWVHILCYC YGLPD---SE PTKTNGKCKS --- | PT Mice **α** | NaTx3.1 | 100 | [5 - 9] |
|  | | Ts5 | KKDGYPVE-- GDNCAFACFG YDNA-YCDKL CKDKKADDGY CVWS-PDCYC YGLPEHILKE PTKTSGRC-- --- | P α | NaTx3.2 | 73 | [10] |
|  | | Tb3 | KKDGYPVE-- ADNCAFVCFG YDNA-YCDKL CGDKKADSGY CYWVHILCYC YGLPD---NE PTKTNGKC-- --- | P Mice α | NaTx3.3 | 88 | [11] |
|  | | TbTx5 | KKDGYPVE-- GDNCAFVCFG YDNA-YCDKL CKDKKADSGY CYWVHILCYC YGLPD---KE PTKTNGRCKP --- | T α | NaTx3.4 | 87 | [12] |
|  | | Tst3 | KKDGYPVE-- YDNCAYICWN YDNA-YCDKL CKDKKADSGY CYWAHITCYC YGLPD---SE PTKTNGKCKS --- | P α | NaTx3.5 | 96 | [13] |
|  | | TdNa8 | KKDGYPVK-- EGDCAFPC-G YDNA-YCDKL CKERKADSGY CYWGNILCYC YGLPD---KA AIKGYGRCRP --- | P*T α | NaTx3.6 | 68 | [2] |
|  | | **Tpa4** | KKDGYPLE-- YDNCAYDCLG YDNK-KCDKL CKDKKADSGY CYWAHILCYC YGLPD---NE PIKTSGRCRP --- | T α | NaTx3.7 | 79 | – |
|  | | **To9** | KKDGYPVK-- EGDCAFPC-G YDNE-YCDKL CKERKADSGY CYWGNILCYC YGLPD---KA AIKGYGRCRP --- | T α | NaTx3.8 | 66 | – |
|  | | **To10** | KKDGYPV--- EGSCAFPC-G YDNA-YCDKL CKERKADSGY CYWVNILCYC YGLPD---NA AIKGYGRCKP --- | T α | NaTx3.9 | 74 | – |
|  | | **To14** | KKDDYPVDTA KRNCMLDCNV WDDEGYCDKF CKGRKADSGY CYKLKAACYC YGLPD---DS PTKTSGRCNP NVR | T α | NaTx3.10 | 59 | – |
|  | |  |  |  |  |  |  |
| **4** | | Ts6 | GREGYPADSK GCKITCFLTA AGYCNTECTL KKGSSGYCAW PACYCYGLPE SVKIWTSETN KC- | P Allerg α | NaTx4.1 | 100 | [14] |
|  | | TsNTxP | GREGYPADSK GCKITCFLTA AGYCNTECTL KKGSSGYCAW PACYCYGLPD SVKIWTSETN KC- | PT Immun α | NaTx4.2 | 98 | [15,16] |
|  | | TbIT-1 | GKEGYPVDSR GCKVTCFFTG AGYCDKECKL KKASSGYCAW PACYCYGLPD SVPVYDNASN KCB | P Ins β | NaTx4.3 | 70 | [17] |
|  | | Tf4 | GKEGYPADSK GCKVTCFFTG VGYCDTECKL KKASSGYCAW PACYCYGLPD SASVWDSATN KC- | P Frog **α** | NaTx4.4 | 77 | [18] |
|  | | TcoNTxP1 | GKEGYPADSK GCKVTCFLTA AGYCNTECKL QKASSGYCAW PACYCYGLPD SASVWDSATN KC- | PT α | NaTx4.5 | 82 | [19] |
|  | |  |  |  |  |  |  |
| **5** | | Ts2 | KEGYAMDHEG CKFSCFIRPA GFCDGYCKTH LKASSGYCAW PACYCYGVPD HIKVWDYATN KC | P Mice β | NaTx5.1 | 100 | [6,20,21] |
|  | | Tst2 | KEGYAMDHEG CKFSCFIRPA GFCDGYCKTH LKASSGYCAW PACYCYGVPD HIKVWDYATN KC | P Mice β | NaTx5.2 | 100 | [11] |
|  | | Tb2 | KEGYAMDHEG CKFSCFPRPA GFCDGYCKTH LKASSGYCAW PACYCYGVPS NIKVWDYATN KC | P β | NaTx5.3 | 95 | [11] |
|  | | Tb2-II | KEGYAMDHEG CKFSCFIRPS GFCDGYCKTH LKASSGYCAW PACYCYGVPS NIKVWDYATN KC | P Mice Ins β | NaTx5.4 | 95 | [17] |
|  | | **To12** | KEGYPMDHEG CKFSCFIRPS GFCERYCKTH LSASTGYCAW PACYCYGVPA NQKVWDYYNN KC | T β | NaTx5.5 | 82 | – |
| Table 2 (*continued*) | | |  |  |  |  |  |
| **Subfamily** | | | **Sequence** | **Charact.** | **NaTx - name** | **% Id.** | **Refs** |
|  | |  |  |  |  |  |  |
| **6** | | Ts1 | -KEGYLMDHE GCKLSCFIRP SGYCGRECGI KKGSSGYCAW PACYCYGLPN WVKVWDRATN KC | P Mice Ins. **β** | NaTx6.1 | 100 | [22 - 25] |
|  | | Tb1/Tb-gamma | -KEGYLMDHE GCKLSCFIRP SGYCGSECKI KKGSSGYCAW PACYCYGLPN WVKVWDRATN KC | PT Mice β | NaTx6.2 | 96 | [11] |
|  | | Tst1/Tst-gamma | GKEGYLMDHE GCKLSCFIRP SGYCGRECTL KKGSSGYCAW PACYCYGLPN WVKVWDRATN KC | PT Mice β | NaTx6.3 | 96 | [11] |
|  | | Tco-gamma | -KEGYAMDHE GCKLSCFIRP SGYCGRECGY KKGSSGYCAW PACYCYGLPN WVKVWERATN RC | T β | NaTx6.4 | 93 | [19] |
|  | |  |  |  |  |  |  |
| **7** | | TdNa9 | -RDGYPQSKV NYCKIYCPNT TVCQWTCKNR AGATDG--DC RWSSCYCFNV APDTVLYGDP GTKPCMA- | PT α | NaTx7.1 | 100 | [2] |
|  | | TdNa10 | -LDGYPLSKN NYCKIYCPNT EVCKDTCKRR AGATDG--EC RWDGCYCFNV APDTKMY--P GELPCH-- | T α | NaTx7.2 | 75 | [2] |
|  | | **Tpa5** | ARDGYPISKN NYCKIYCPNT KVCKETCKNR ASAPDGECDG -WNLCYCFKV PDNIPVWGDP GTPPCMT- | T α | NaTx7.3 | 67 | – |
|  | | **Tpa6** | ARDGYPLSKN NNCKIYCPDT DVCKDTCKNR ASAPDGKCDG -WNSCYCFKV PDHIPVWGDP GTKPCMT- | T α | NaTx7.4 | 67 | – |
|  | | **To6** | -LDGYPLSKN NYCKIYCPDE KVCKWSCKHR AGATNGKGDC INKGCYCYDV APGTEMY--P GRLPCNPY | PT α | NaTx7.5 | 60 | [26] – |
|  | | **To7** | -LDGYPLSKI NNCKIYCPDD DVCKWTCKHR AGATNGKGDC IWYGCYCYDV APGTKMY--P GSSPCYA- | PT α | NaTx7.6 | 64 | [26] – |
|  | |  |  |  |  |  |  |
| **8** | | **To13** | IKNGYPRDSK GCTFECGQDA KHGDDYCDKM CKTTLKGEGG DCDFEYAECW CDNIPDTVVT WKNKEPKCKQ I | T | NaTx8.1 | 100 | – |
|  | |  |  |  |  |  |  |
| **9** | | **Tpa8** | LKNGYPVIEG GGSPDYGESA ECGSEDSNSA DNFCNDICTN VGGKSGDCCL GSCFCFDLPD EQKTVEVMDR TKEYCEFVE | T | NaTx9.1 | 100 | – |
|  | |  |  |  |  |  |  |
| **10** | | **To8** | KEGYLLGSRG CKMNCLTRPE KFCELECSLV GGENGYCAYW LACYCYNVPE SVKLWESDTN EC | PT β | NaTx10.1 | 100 | [26] – |
|  | |  |  |  |  |  |  |
| **11** | | **Tc48a** | NKDGYLMEGD GCKMGCLTRK ASYCVDQCKE VGGKDGYCYA WLSCYCYNMP DSVEIWDSKN NKCGK | PT **α’** | NaTx11.1 | 100 | [26] |
|  | |  |  |  |  |  |  |
| **12** | | **Tc49b** | KKEGYLVGND GCKYGCITRP HQYCVHECEL KKGTDGYCAY WLACYCYNMP DWVKTWSSAT NKCK- | PT not **α** | NaTx12.1 | 100 | [27] |
|  | | **Tpa2** | KKEGYLVGND GCKYSCFTRP AQYCVHECEL RKGTDGYCYA WLACYCYNMP DHVRTWSRAT NRCGS | P **β** | NaTx12.2 | 82 | [28] |
|  | |  |  |  |  |  |  |
| **13** | | Td1 | KDGYLMEPNG CKRGCLTRPA RYCPNECSRL KGKDGYCYLW LACYCYNMPE SAPVWERATN RCGK | PT β | NaTx13.1 | 100 | [1] |
|  | | Td2 | KEGYLMGADG CKRSCLTRPG HYCANECSRV KGTDGYCYAW LACYCYNMPN WVKTWDRATN TCGR | P*T β | NaTx13.2 | 70 | [1] |
|  | | Td3 | KDGYLMGPDG CKLDCLMRKG TFCAETCSLR KGKDGYCYAW LACYCYNMPD SVKVWERATN RCGK | P*T β | NaTx13.3 | 71 | [1] |
|  | | Td6 | KEGYLMEANG CKRSCTLRPG HYCANECSYV KGKNGYCYAW VACYCYNMPD SVKIWDSATN TCGR | T β | NaTx13.4 | 67 | [1] |
|  | | Td10 | KDGYLMGPDG CKRGCLTRPA RYCPNECSRL KGKDGYCYLW LACYCYNMPE SAPVWERATN RCGK | T β | NaTx13.5 | 96 | [1] |
|  | | Td11 | KDGYLMGSDG CKLDCLMKKG TYCADECSRV KGKDGYCYLW LACYCYNMPD SVKVWERATN RCGK | T β | NaTx13.6 | 75 | [1] |
|  | | Td12 | KDGYLMEPNG CKRGCLTRPA RYCANECSRV KGTDGYCYAW LACYCYNMPN WVKTWDRATN TCGR | T β | NaTx13.7 | 81 | [1] |
|  | | TdNa7 | KDGYLMGPDG CKLDCLMRKG TFCAETCSLR KGKDGYCYAW LACYCYNMPD WVKTWERATN TCGK | PT β | NaTx13.8 | 67 | [2] |
|  | | **To4** | KDGYLMEYGG CKMSCLMKKG TFCAEECTRM KGKDGYCYAW LACYCYNMPD WVKIWNRATN KC-- | PT β | NaTx13.9 | 64 | [27] – |
|  | |  |  |  |  |  |  |
| **14** | | Tz1 | KDGYLVGNDG CKYSCFTRPG TYCANECSRV KGKDGYCYAW MACYCYSMPN WVKTWDRATN RCGR | PT **β** | NaTx14.1 | 100 | [29] |
|  | | **Tc48b/Tc49a** | KDGYLVGNDG CKYNCLTRPG HYCANECSRV KGKDGYCYAW MACYCYSMPD WVKTWSRSTN RCGR | PT **α’** | NaTx14.2 | 90 | [30] |
|  | | Td4 | KDGYLVGNDG CKYSCFTRPG TYCANECSRV KGKDGYCYAW MACYCYSMPN WVKTWDRATN RCGR | P*T β | NaTx14.3 | 100 | [1] |
|  | | Td5 | KDGYLVGNDG CKYSCSTRPG HYCASECSRV KGKDGYCYAW LACYCYNMPN WAPIWNSATN RCR- | P*T β | NaTx14.4 | 82 | [1] |
|  | | Td7 | KDGYLVGADG CKYGCFTRPG HFCASECSLL KGKDGYCYAW LACYCYNLPD SVPVWDSATN RCGK | T β | NaTx14.5 | 75 | [1] |
|  | | Td8 | KDGYLVGDDG CKMHCFTRPG HYCASECSRV KGKDGYCYAW LACYCYNMPN WAPIWNSATN RCR- | PT β | NaTx14.6 | 79 | [1] |
|  | | Td9 | KDGYLVGDDG CKMHCFTRPG HYCASECSRV KGKDGYCYAW LACYCYNMPN WAPIWNSATN SCGK | T β | NaTx14.7 | 78 | [1] |
|  | | Bactridin-2 | KDGYLVGNDG CKYSCFTRPG TYCANECSRV KGKDGYCYAW MACYCYSMPN WVKTWNRATN RCGR | P Mice Antm β | NaTx14.8 | 98 | [4] |
|  | | **To11** | KDGYLVGNDG CKYNCLTRPG HYCANECSRV KGKDGYCYAW MACYCYNMPN WVKTWSRATN KC-- | T | NaTx14.9 | 90 | – |

**References**

**1. Borges A, García CC, Lugo E, Alfonzo MJ, Jowers MJ, et al. (2006) Diversity of long-chain toxins in *Tityus zulianus* and *Tityus discrepans* venoms (Scorpiones, Buthidae): molecular, immunological, and mass spectral analyses. Comp Biochem Physiol C Toxicol Pharmacol 142: 240-252.**

**2. D'Suze G, Schwartz EF, García-Gómez BI, Sevcik C, Possani LD (2009) Molecular cloning and nucleotide sequence analysis of genes from a cDNA library of the scorpion *Tityus discrepans*. Biochimie 91: 1010-1019.**

**3. D'Suze G, Sevcik C, Corona M, Zamudio FZ, Batista CV, et al. (2004) Ardiscretin a novel arthropod-selective toxin from *Tityus discrepans* scorpion venom. Toxicon 43: 263-272.**

**4. Díaz P, D'Suze G, Salazar V, Sevcik C, Shannon JD, et al. (2009) Antibacterial activity of six novel peptides from *Tityus discrepans* scorpion venom. A fluorescent probe study of microbial membrane Na^+^ permeability changes. Toxicon 54: 802-817.**

**5. Kirsch GE, Skattebol A, Possani LD, Brown AM (1989) Modification of Na^+^ channel gating by an alpha scorpion toxin from *Tityus serrulatus*. J Gen Physiol 93: 67-83.**

**6. Possani LD, Martin BM, Fletcher MD, Fletcher PLJ (1991) Discharge effect on pancreatic exocrine secretion produced by toxins purified from *Tityus serrulatus* scorpion venom. J Biol Chem 266: 3178-3185.**

**7. Corona M, Zurita M, Possani LD, Becerril B (1996) Cloning and characterization of the genomic region encoding toxin IV-5 from the scorpion *Tityus serrulatus* Lutz and Mello. Toxicon 34: 251-256.**

**8. Campos FV, Moreira TH, Beirão PS, Cruz JS (2004) Veratridine modifies the TTX-resistant Na^+^ channels in rat vagal afferent neurons. Toxicon 43: 401-406.**

**9. Campos FV, Coronas FI, Beirão PS (2004) Voltage-dependent displacement of the scorpion toxin Ts3 from sodium channels and its implication on the control of inactivation. Br J Pharmacol 142: 1115-1122.**

**10. Marangoni S, Toyama MH, Arantes EC, Giglio JR, da Silva CA, et al. (1995) Amino acid sequence of TsTX-V, an alpha-toxin from *Tityus serrulatus* scorpion venom, and its effect on K^+^ permeability of beta-cells from isolated rat islets of Langerhans. Biochim Biophys Acta 1243: 309-314.**

**11. Becerril B, Corona M, Coronas FI, Zamudio F, Calderón-Aranda ES, et al. (1996) Toxic peptides and genes encoding toxin gamma of the Brazilian scorpions *Tityus bahiensis* and *Tityus stigmurus*. Biochem J 313 ( Pt 3): 753-760.**

**12. Kalapothakis E, Jardim S, Magalhães AC, Mendes TM, De Marco L, et al. (2001) Screening of expression libraries using ELISA: identification of immunogenic proteins from *Tityus bahiensis* and *Tityus serrulatus* venom. Toxicon 39: 679-685.**

**13. Batista CV, Román-González SA, Salas-Castillo SP, Zamudio FZ, Gómez-Lagunas F, et al. (2007) Proteomic analysis of the venom from the scorpion *Tityus stigmurus*: biochemical and physiological comparison with other *Tityus* species. Comp Biochem Physiol C Toxicol Pharmacol 146: 147-157.**

**14. Marangoni S, Ghiso J, Sampaio SV, Arantes EC, Giglio JR, et al. (1990) The complete amino acid sequence of toxin TsTX-VI isolated from the venom of the scorpion *Tityus serrulatus*. J Protein Chem 9: 595-601.**

**15. Chávez-Olórtegui C, Kalapothakis E, Ferreira AM, Ferreira AP, Diniz CR (1997) Neutralizing capacity of antibodies elicited by a non-toxic protein purified from the venom of the scorpion *Tityus serrulatus*. Toxicon 35: 213-221.**

**16. Guatimosim SC, Prado VF, Diniz CR, Chávez-Olórtegui C, Kalapothakis E (1999) Molecular cloning and genomic analysis of TsNTxp: an immunogenic protein from *Tityus serrulatus* scorpion venom. Toxicon 37: 507-517.**

**17. Pimenta AM, Martin-Eauclaire M, Rochat H, Figueiredo SG, Kalapothakis E, et al. (2001) Purification, amino-acid sequence and partial characterization of two toxins with anti-insect activity from the venom of the South American scorpion *Tityus bahiensis* (Buthidae). Toxicon 39: 1009-1019.**

**18. Wagner S, Castro MS, Barbosa JA, Fontes W, Schwartz EF, et al. (2003) Purification and primary structure determination of Tf4, the first bioactive peptide isolated from the venom of the Brazilian scorpion *Tityus fasciolatus*. Toxicon 41: 737-745.**

**19. Diego-García E, Batista CV, García-Gómez BI, Lucas S, Candido DM, et al. (2005) The Brazilian scorpion *Tityus costatus* Karsch: genes, peptides and function. Toxicon 45: 273-283.**

**20. Sampaio SV, Arantes EC, Prado WA, Riccioppo Neto F, Giglio JR (1991) Further characterization of toxins T1IV (TsTX-III) and T2IV from *Tityus serrulatus* scorpion venom. Toxicon 29: 663-672.**

**21. Mansuelle P, Martin-Eauclaire MF, Chávez-Olórtegui C, de Lima ME, Rochat H, et al. (1992) The beta-type toxin Ts II from the scorpion *Tityus serrulatus*: amino acid sequence determination and assessment of biological and antigenic properties. Nat Toxins 1: 119-125.**

**22. Martin-Eauclaire MF, Ceard B, Ribeiro AM, Diniz CR, Rochat H, et al. (1992) Molecular cloning and nucleotide sequence analysis of a cDNA encoding the main beta-neurotoxin from the venom of the South American scorpion *Tityus serrulatus*. FEBS Lett 302: 220-222.**

**23. Bechis G, Sampieri F, Yuan PM, Brando T, Martin MF, et al. (1984) Amino acid sequence of toxin VII, a beta-toxin from the venom of the scorpion *Tityus serrulatus*. Biochem Biophys Res Commun 122: 1146-1153.**

**24. Vijverberg HP, Pauron D, Lazdunski M (1984) The effect of *Tityus serrulatus* scorpion toxin gamma on Na^+^ channels in neuroblastoma cells. Pflugers Arch 401: 297-303.**

**25. Yatani A, Kirsch GE, Possani LD, Brown AM (1988) Effects of New World scorpion toxins on single-channel and whole cell cardiac sodium currents. Am J Physiol 254: H443-451.**

**26. Batista CV, del Pozo L, Zamudio FZ, Contreras S, Becerril B, et al. (2004) Proteomics of the venom from the Amazonian scorpion *Tityus cambridgei* and the role of prolines on mass spectrometry analysis of toxins. J Chromatogr B Analyt Technol Biomed Life Sci 803: 55-66.**

**27. Batista CV, Zamudio FZ, Lucas S, Fox JW, Frau A, et al. (2002) Scorpion toxins from *Tityus cambridgei* that affect Na^+^-channels. Toxicon 40: 557-562.**

**28. Barona J, Batista CV, Zamudio FZ, Gomez-Lagunas F, Wanke E, et al. (2006) Proteomic analysis of the venom and characterization of toxins specific for Na^+^ - and K^+^ -channels from the Colombian scorpion *Tityus pachyurus*. Biochim Biophys Acta 1764: 76-84.**

**29. Borges A, Alfonzo MJ, García CC, Winand NJ, Leipold E, et al. (2004) Isolation, molecular cloning and functional characterization of a novel beta-toxin from the Venezuelan scorpion, *Tityus zulianus*. Toxicon 43: 671-684.**

**30. Murgia AR, Batista CV, Prestipino G, Possani LD (2004) Amino acid sequence and function of a new alpha-toxin from the Amazonian scorpion *Tityus cambridgei*. Toxicon 43: 737-740.**
